# HIV-1 genetic diversity and antiretroviral drug resistance among individuals from Roraima state, northern Brazil

**DOI:** 10.1371/journal.pone.0173894

**Published:** 2017-03-16

**Authors:** André de Lima Guerra Corado, Gonzalo Bello, Renato Augusto Carvalho Leão, Fabiana Granja, Felipe Gomes Naveca

**Affiliations:** 1 Instituto Leônidas e Maria Deane, FIOCRUZ, Manaus, Amazonas, Brazil; 2 Laboratório de AIDS e Imunologia Molecular, Instituto Oswaldo Cruz, FIOCRUZ, Rio de Janeiro, Brazil; 3 Laboratório Central de Saúde Pública de Roraima (LACEN/RR), Boa Vista, Roraima, Brazil; 4 Laboratório de Biologia Molecular, Universidade Federal de Roraima (UFRR), Boa Vista, Roraima, Brazil; National and Kapodistrian University of Athens, GREECE

## Abstract

The HIV-1 epidemic in Brazil has spread towards the Northern country region, but little is known about HIV-1 subtypes and prevalence of HIV strains with resistance mutations to antiretrovirals in some of the Northern states. HIV-1 protease (PR) and reverse transcriptase (RT) sequences were obtained from 73 treatment-naive and -experienced subjects followed between 2013 and 2014 at a public health reference unit from Roraima, the northernmost Brazilian state. The most prevalent HIV-1 clade observed in the study population was the subtype B (91%), followed by subtype C (9%). Among 12 HIV-1 strains from treatment-naïve patients, only one had a transmitted drug resistance mutation for NNRTI. Among 59 treatment-experienced patients, 12 (20%) harbored HIV-1 strains with acquired drug resistance mutations (ADRM) that reduce the susceptibility to two classes of antiretroviral drugs (NRTI and NNRTI or NRTI and PI), and five (8%) harbored HIV-1 strains with ADRM that reduced susceptibility to only one class of antiretroviral drugs (NNRTI or PI). No patients harboring HIV strains with reduced susceptibility to all three classes of antiretroviral drugs were detected. A substantial fraction of treatment-experienced patients with (63%) and without (70%) ADRM had undetectable plasma viral loads (<40 copies/ml) at the time of sampling. Among treatment-experienced with plasma viral loads above 2,000 copies/ml, 44% displayed no ADRM. This data showed that the HIV-1 epidemic in Roraima displayed a much lower level of genetic diversity and a lower prevalence of ADRM than that described in other Brazilian states.

## Introduction

In the early of 1980s the first case of HIV-1 infection was officially diagnosed in Brazil and by the end of 2014, about 780,000 people were living with HIV/AIDS in this country [1]. During the first 15 years of the epidemic, most AIDS cases were concentrated in the capital cities of the Southern and Southeastern regions, but since 1995 there was a clear expansion of the AIDS cases to the capital and inner cities of the Northeastern, Central-Western and Northern Brazilian regions [1]. HIV-1 subtype B accounts for most (70%) of HIV infections in Brazil, although other genetic variants including subtype F1, subtype C, and intersubtype recombinants (B/F and B/C) can also be found at relatively high prevalences around the country [2–8].

Nearly half (52%) of Brazilian people living with HIV/AIDS are under antiretroviral therapy (ART) [1]. This has substantially reduced the AIDS-related morbidity and mortality, but also has led to a substantial increase in the emergence and transmission of drug resistant viruses in the Brazilian population [9]. Nation-wide studies of HIV-infected treatment-naive individuals estimate that the overall rate of transmitted drug resistance mutations (TDRM) to nucleoside reverse transcriptase inhibitors (NRTI), non-nucleoside reverse transcriptase inhibitors (NNRTI) and/or protease inhibitors (PI) in Brazil increased from 6.6% to 12.2% over the past 10–15 years [2–5,10]. In the same period, the prevalence of acquired drug resistance mutations (ADRM) to NRTI, NNRTI and/or PI remained persistently high (>80%) among ART-experienced patients from Brazil [11–19].

Brazil is a continental sized country and information about the prevalence of HIV-1 subtypes and antiviral drug resistance mutations in some locations away from the most populated urban centers, particularly in the Northern Brazilian region, are scarce. Roraima is the northernmost and least populated state of Brazil, with about 500,000 inhabitants in 2015, mostly concentrated (65%) in the state capital Boa Vista. Roraima is bordered by Venezuela to the north and northwest, Guyana to the east, the state of Amazonas to the south and southwest and the state of Para to the southeast. By June 2015, 1,821 AIDS cases had been cumulatively reported in Roraima since the first identification of AIDS in the state in 1988, most of them among heterosexuals (56%) and male (66%) individuals [1].

Roraima represents an interesting location for molecular surveillance studies of the HIV epidemic because of its geographic localization and AIDS incident rate trends. Roraima maintains a very intense movement of people with Venezuela and Guyana owing to activities such as tourism, sexual exploitation and mining [20], nowadays increased by the economic crisis in Venezuela, supporting for the possibility of an intense HIV flux with those neighboring countries. Moreover, the incidence rate of AIDS cases in Roraima (29.2/100,000 inhabitants) and its capital Boa Vista (37.8/100,000 inhabitants) was well above the national mean (19.7 cases per 100.000 inhabitants) in 2014 [1]. Roraima currently figures among the top five states with the country's largest incidence rates of AIDS cases, supporting the dynamic nature of the HIV epidemic in this Brazilian state.

The only HIV molecular epidemiologic study performed in Roraima revealed that subtype B was the predominant viral strain (86%), followed by BF recombinant variants (14%) [8]. That study, however, analyzed a very low number of patients (*n* = 36) and with no description about the prevalence of drug resistance mutations among the study population. The objective of this study was to perform a cross-sectional study of the HIV-1 subtypes and rate of TDRM and ADRM detected among HIV-infected patients from different regions of the Roraima state.

## Materials and methods

### Study patients

We collected 5ml of peripheral blood samples from 73 HIV-1-infected persons that attended the Public Health Central Laboratory in Boa Vista/Roraima (LACEN-RR), a reference unit from the Brazilian Ministry of Health that receives samples for monitoring of CD4+ T cell count and plasma viral load, between January 2013 and February 2014. Inclusion criteria were adult patients (≥18 years of age) of either sex with a recent or chronic diagnosis of HIV-1 infection, and of any treatment status. Our sampling represents more than 10% of the HIV-infected population in Roraima in this period and covered eight out of 15 municipalities in the Roraima state. All patients that participated in this study were informed of the procedures and signed the informed consent. This study was approved by the Ethics Research Committee of the Federal University of Roraima (COEP/UFRR) (CAAE number: 15629013.8.0000.5302).

### HIV-1 DNA amplification and sequencing

Blood samples collected in Boa Vista were transported to the Instituto Leônidas e Maria Deane, the FIOCRUZ unit in Manaus, for HIV amplification and sequencing. Genomic DNA was extracted from 200 μl of total blood using the automated system QIAcube, with QIAamp DNABlood Kit (QIAGEN, Germany), following manufacturer’s recommendations. The entire protease (PR) and part of the reverse transcriptase (RT) (nucleotides 2253–3272 relative to the HXB2 clone) gene was amplified and sequenced as previously described [21]. Sequences were assembled using the Geneious software package version 7.1.4 (Biomatters Limited, Auckland, New Zealand).

### HIV-1 subtyping

HIV-1 subtypes were initially determined with the REGA HIV-1 Subtyping Tool 3.0 software [22] and later confirmed by phylogenetic and bootscanning analyses with HIV-1 reference sequences of subtypes A–D, F–H, J, and K retrieved from the Los Alamos HIV database (www.hiv.lanl.gov). Maximum-likelihood (ML) phylogenetic trees were reconstructed with the PhyML 3.0 program [23,24] to verify the clustering of Brazilian HIV-1 sequences with subtypes, using the approximate likelihood-ratio test (a*LRT*) [25] to estimate the reliability of the obtained tree topology. All sequences were also subjected to bootscanning analyses using the SimPlot 3.5.1 software [26] to identify possible recombination breakpoints. Bootstrap values supporting branching with HIV-1 reference sequences were determined in Neighbor Joining (NJ) trees, based on 100 resamplings, with a 250 nt sliding window moving in steps of 10 bases.

### HIV-1 drug resistance analyses

The TDRM and ADRM were identified using the Calibrated Population Resistance (CPR) tool [27] (http://cpr.stanford.edu/cpr.cgi) and the Genotypic Resistance Interpretation Algorithm of the HIVdb program [28] (http://sierra2.stanford.edu/sierra/servlet/JSierra), respectively, both available through the Stanford University HIV Drug Resistance Database. The HIVdb program was also used to infer the resistance profile of the HIV-1 sequences.

### Nucleotide sequence accession numbers

HIV-1 *pol* (PR/RT) sequences from Roraima were deposited in GenBank under accession numbers KX443015-KX443087.

## Results

The epidemiological characteristics of the study population are described in Table 1 (individual data for each patient is described in the S1 Table). Among the 73 individuals included in this study, 12 were drug naïve and 61 were under ART. The period of diagnosis of the HIV infection ranged between 1995 and 2013. Most patients were males (57.5%) and the overall median age was 37 years (21–57 years range), with similar values for both males (37 years, 24–57 years range) and females (37 years, 21–56 years range). More male patients declared to be heterosexual (57%) than homosexual (33%) or bisexual (10%), whereas only one female declared to be homosexual. The major risk factor for HIV infection identified was unprotected sex (81%), followed by iatrogenic transmission/intravenous drug use (12%), while for 7% of the patients the route of transmission was unknown. The median of CD4 T cell counts was 448 cells/μl (74–1,822 cells/μl range). The CD4 T cell counts was <350 cells/μl for 36%, between 350 and 500 cells/μl for 22%, and >500 cells/μl for 33% of the participants. The median of plasma viral load was 54,614 copies/ml (<40–1,383,017 copies/ml range). Plasma viral loads were below limit of detection (40 copies/ml) for 58%, between 51 and 10,000 copies/ml for 19%, and over 10,000 copies/ml for 16% of the participants. Most patients (79%) were from the capital, Boa Vista. The remaining ones were from other municipalities of the Roraima state (16%) or neighboring cities outside Roraima (5%), including one patient from Manaus (Amazonas state), two patients from St. Helena de Uairén (Venezuela) and one patient from Lethem (Guyana).

**Table 1:** Epidemiological and clinical characteristics of HIV-1-infected patients attended at the Public Health Central Laboratory in Boa Vista, Roraima.

	All (n = 73)	ART-naïve (n = 12)	ART-experienced (n = 61)
**Gender**			
Male	42 (57.5%)	4 (33.3%)	38 (62.3%)
Female	31 (42.5%)	8 (66.4%)	23 (37.7%)
**Age Median (Range)**	37 (21–57)	35 (21–46)	38 (22–57)
**CD4 T cells**			
<350 cells/l	26 (35.6%)	3 (25.0%)	23 (37.7%)
350–500 cells/ul	16 (21.9%)	2 (16.7%)	14 (23.0%)
>500 cells/ul	24 (32.9%)	4 (33.3%)	20 (32.7%)
Unknown	7 (9.6%)	3 (25.0%)	4 (6.6%)
**HIV-1 RNA viral load**			
<40 copies/ml	42 (57.5%)	2 (16.7%)	40 (65.6%)
50–10,000 copies/ml	14 (19.2%)	1 (8.3%)	13 (21.3%)
>10,000 copies/ml	12 (16.4%)	6 (50.0%)	6 (9.8%)
Unknown	5 (6.8%)	3 (25.0%)	2 (3.3%)
**HIV-1 diagnosis (year)**			
1995–1999	3 (4.1%)	-	3 (4.9%)
2000–2004	12 (16.4%)	1 (8.3%)	11 (18.0%)
2005–2009	15 (20.5%)	2 (16.6%)	13 (21.3%)
2010–2013	41 (56.2%)	9 (75.1%)	32 (52.5%)
Unknown	2 (2.7%)	-	2 (3.3%)
**Route of infection**			
Heterosexual	40 (54.8%)	10 (83.4%)	30 (49.2%)
MSM	19 (26.0%)	-	19 (31.1%)
Blood contact	9 (12.3%)	1 (8.3%)	8 (13.1%)
Unknown	5 (6.8%)	1 (8.3%)	4 (6.6%)
**Origin**			
Boa Vista	58 (79.5%)	11(91.6%)	47 (77.0%)
Bonfim	3 (4.1%)	-	3 (4.9%)
Rorainópolis	3 (4.1%)	-	3 (4.9%)
Others^a^	5 (6.8%)	-	5 (8.1%)
Manaus	1 (1.4%)	-	1 (1.7%)
Santa Helena do Uairén	2 (2.7%)	1 (8.4%)	1 (1.7%)
Lethem	1 (1.4%)	-	1 (1.7%)

^a^ Cantá, Caracaraí, Pacaraima, São Luis do Anauá and Alto Alegre municipalities

**Fig 1:**
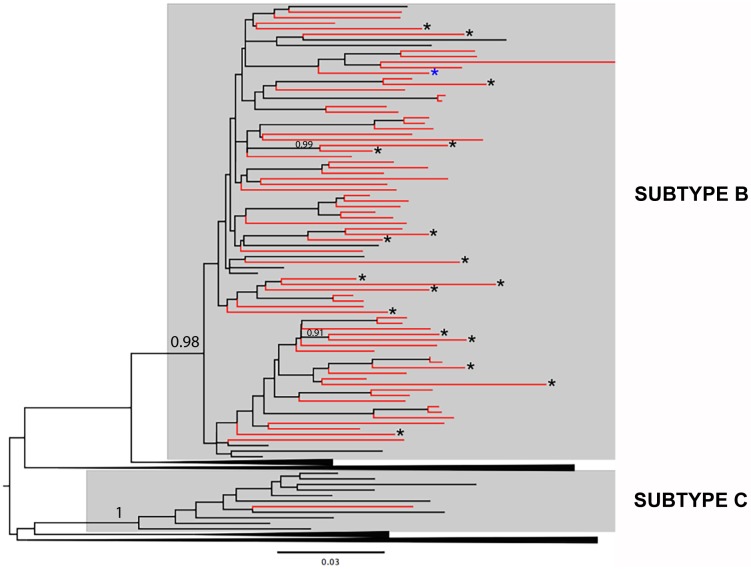
Maximum likelihood phylogenetic tree of HIV-1 *pol* (PR/RT) sequences obtained from ART naive and experienced patients from the Roraima state. HIV-1 *pol* sequences from Roraima (red terminal branches) were combined with reference sequences (black terminal branches) of all HIV-1 group M subtypes (A–D, F–H, J, and K). Shaded boxes indicate the position of HIV-1 subtypes detected in Roraima. Asterisks indicate the position of sequences with ADRM (dark) and TDRM (blue). Only a*LRT* values at the branch of subtype B, subtype C, and the two highly supported clades of sequences with ADRM are shown. Trees are rooted at midpoint and the branch lengths are drawn to scale with the bar at the bottom indicating nucleotide substitutions per site.

All patients recruited had HIV-1 *pol* (PR/RT) genes amplified and sequenced, and nearly all were characterized as subtype B (91%), except one classified as subtype C (9%) (Fig 1). Among the 12 treatment-naïve patients analyzed, only one harbored a TDRM to NNRTI (G190A), whereas no TDRM to NRTI or PI were detected in the study population. Two out of 61 HIV-1 PR/RT sequences obtained from patients under ART were excluded due to the presence of APOBEC3GF-mediated G to A mutations. Among the remaining 59 treatment-experienced patients, 17 (29%) harbored HIV-1 strains with ADRM that conferred some level of resistance to NNRTI, NRTI and/or PI (Table 2). Among these, 14 (24%) patients had ADRM to NNRTI (being K103N and G190A the most common), 12 (20%) patients had ADRM to NRTI (being M184V the most frequently found) and three (5%) patients had ADRM to PI. Five (8%) patients showed ADRM to only one class of antiretroviral drugs (NNRTI or PI), 10 (17%) patients showed ADRM to both NRTI and NNRTI, and two (3%) patients showed ADRM to both NRTI and PI. No patients showed ADRM to all three classes of antiretroviral drugs. The median plasma viral load of patients without ADRM (2,917 copies/ml) was much lower than of those with ADRM (130,360 copies/ml range), although the proportion of patients without ADRM that displayed undetectable plasma viral loads (70%) was similar to the corresponding proportion of patients with ADRM (63%) and the prevalence of ADRM among patients with detectable plasma viral loads (32%) was only slightly higher than among patients with undetectable viral loads (25%). HIV-1 sequences containing DRM were widely dispersed in the phylogenetic tree among sequences with no DRM (Fig 1). Only two highly supported (a*LRT* > 0.90) pairs of related sequences with ADRM were identified (Fig 1), but sequences within each pair displayed a different set of ADRM (Table 2).

**Table 2:** List of mutations known to confer reduced susceptibility to antiretroviral agents among ART-experienced HIV-1-infected patients attended at the Public Health Central Laboratory in Boa Vista, Roraima.

Patient	PI major mutations	NRTI mutations	NNRTI mutations	Resistance profile		
				Low	Intermediate	High
BR.RR.2013.RLMDS12	-	-	E138A	ETR, RPV	-	-
BR.RR.2013.MG29	M46I, I54V, V82A, L90M	D67N, T69D, K70R, M184V, K219Q	-	TDF	TPV, ABC, AZT, D4T	ATV, FPV, IDV, LPV, NFV, SQV, 3TC, DDI, FTC
BR.RR.2013.JDOG35^a^	-	T69N, M184V, K219R	K103N, P225H	ABC, DDI	-	3TC, FTC, EFV, NVP
BR.RR.2013.VFDB50	-	M184V	K103N	ABC, DDI	-	3TC, FTC, EFV, NVP
BR.RR.2013.FSDC52^a^	-	-	K103N, G190R	-	-	EFV, NVP
BR.RR.2013.ACDC56	-	-	K103N	-	-	EFV, NVP
BR.RR.2013.CDSF64	-	M184V	V179D	ABC, DDI, EFV, ETR, NVP, RPV	-	3TC, FTC
BR.RR.2013.RFDS66	V32I, V82A	M184V	-	DRV, SQV, ABC, DDI	ATV, FPV, IDV, LPV, NFV,	3TC, FTC
BR.RR.2013.VD71	-	M41L, M184V, L210W, T215Y	K103N, Y188L	ETR	TDF	3TC, ABC, AZT, D4T, DDI, FTC, EFV, NVP, RPV
BR.RR.2013.OVM76	-	M184V	K103N, V108I, K238T	ABC, DDI	-	3TC, FTC, EFV, NVP
BR.RR.2013.SA79	Q58E^b^	-	-	TPV	-	-
BR.RR.2013.TDSA85	-	M184V, T215Y	K103S, G190A	DDI, ETR, RPV	ABC, AZT, D4T,	3TC, FTC, EFV, NVP
BR.RR.2013.LRD88	-	M184V	K103N	ABC, DDI	-	3TC, FTC, EFV, NVP
BR.RR.2013.ION94^a^	-	D67N, T69D, K70R, M184V, T215F, K219Q	K101H, K103N, G190A	ETR, RPV	TDF	3TC, ABC, AZT, D4T, DDI, FTC, EFV, NVP
BR.RR.2013.FSDC99	-	-	E138A	ETR, RPV	-	-
BR.RR.2013.DJSG100	-	D67N, T69N, K70R, M184V, K219Q	V90I, K103N, Y188L	TDF, ETR	ABC, D4T, DDI	3TC, AZT, FTC, EFV, NVP, RPV
BR.RR.2013.ECV106^a^	-	M184V	K103N, V108I	ABC, DDI	-	3TC, FTC, EFV, NVP

^a^ Sequence pairs BR.RR.2013.JDOG35/BR.RR.2013.ION94 and BR.RR.2013.FSDC52/ BR.RR.2013.ECV106 branched in highly supported monophyletic clusters in ML analysis.

^b^ PI minor resistance mutation.

ABC: abacavir; ATV: atazanavir; AZT: zidovudine; DDI: didanosine; DLV: delavirdine; D4T: stavudine; EFV: efavirenz; ETR: etravirine; FTC: emtricitabine; FPV: fosamprenavir; IDV: indinavir; LPV: lopinavir; NFV: nelfinavir; NVP: nevirapine; RPV: rilpivirine; SQV: saquinavir; TDF: tenofovir; TPV: tipranavir and 3TC: lamivudine.

## Discussion

The current analysis showed that the HIV-1 epidemic in the northernmost Brazilian state of Roraima is almost exclusively driven by the subtype B. Among the 73 HIV-1 *pol* (PR/RT) gene sequences analyzed, 72 were classified as subtype B and one as subtype C. A previous study analyzed 36 HIV-1 *pol* (PR/RT) gene sequences from Roraima and classified 31 sequences as subtype B and five sequences as BF recombinants [8]. When both studies are combined, we obtained that the predominant HIV-1 clade circulating in Roraima was subtype B (94%), followed by BF recombinants (5%) and subtype C (1%).

These results indicate that the HIV-1 molecular epidemiologic profile observed in Roraima is much more homogenous than that described in any other Brazilian state, even from the Northern region [8,18,29–33], but comparable to that described in the neighboring country of Venezuela [34,35]. Roraima is one of the most isolated Brazilian states, being connected by land only to the state of Amazonas. On the other hand, Boa Vista (the capital of the Roraima state) is located close to the Venezuelan and Guyanese cities of St. Helena de Uairén (230 km) and Lethem (114 km), respectively. Notably, among the 73 patients included in our study and that attended the Public Health Central Laboratory in Boa Vista, two patients were from St. Helena de Uairén and one patient from Lethem, reflecting the intense human mobility across the Roraima borders with Venezuela and Guyana. Consistent with this, we recently described a singular high prevalence of non-pandemic subtype B variants of Caribbean origin in Roraima [36]. These data suggest that the HIV-1 epidemic in Roraima is more closely related to epidemics in neighboring South American countries (Venezuela and Guyana) than to epidemics in other Brazilian regions.

Among the 12 HIV-1 *pol* sequences from drug naïve patients here analyzed, only one displayed a TDRM to NNRTI, whereas no TDRM to NRTI or PI were detected. A much higher level of ADRM (28%), by contrast, was observed among subjects under ART included in our study. Most HIV-1 isolates with ADRM harbored some level of resistance to both NRTI and NNRTI (60%), whereas others displayed resistance to only NNRTI (23%), to both PI and NRTI (12%), or to only PI (6%). No patients with ADRM to all three classes of antiretroviral drugs were detected in this study. The phylogenetic analysis reveals no highly supported clades of HIV-1 sequences with the same set of ADRM, thus supporting that DRM observed among patients under ART most probably evolved independently after initiation of therapy and were not directly transmitted among subjects.

The prevalence of ADRM here detected in Roraima (28%) was relatively low compared to the higher levels of resistance to NRTI, NNRTI and/or PI (>80%) reported in other Brazilian states from all country regions [11–19]. It could be argued that most previous studies of ADRM conducted in Brazil analyzed populations of HIV-1-infected individuals failing ART and that the relative low rate of ADRM observed in Roraima may be explained by a low frequency of virological failure among treated patients. However, the estimated prevalence of ADRM in Roraima remains comparatively lower to that described in other Brazilian states even if we only considered those ART-experienced patients with detectable viral load (32%), or those patients with plasma viral loads >1,000 copies/ml (56%) that is the viral load cut-off for HIV resistance genotyping according to the guidelines of the Brazilian Ministry of Health (http://www.aids.gov.br/pagina/2012/51642).

It is also interesting to note that although the median plasma viral load of patients without ADRM (2,917 copies/ml) was much lower than of those with ADRM (130,360 copies/ml range), not all patients harboring ADRM displayed evidence of treatment failure. Indeed, a significant proportion of patients with ADRM (63%) displayed undetectable plasma viral loads at the time of sampling, and the prevalence of ADRM among patients with detectable plasma viral loads (32%) was only slightly higher than among patients with undetectable viral loads (25%). On the other hand, among the ART-experienced patients with plasma viral loads >1,000 copies/ml, 44% harbored HIV strains fully susceptible to antiretrovirals. Thus, a significant proportion of the apparent therapeutic failures among HIV-1-infected patients from Roraima could probably be attributed to problems of sub-optimal therapeutic adherence rather than to selection of drug-resistance strains.

Although this study suggests a low prevalence of both TDRM and ADRM among HIV-infected individuals from the Roraima state, these results should be interpreted with caution due to the relatively low size of the study populations. New surveillance studies of both untreated and ART-treated patients will be necessary to obtain more accurate estimates of the prevalence of TDRM and ADRM in this Brazilian state. The application of more specific questionnaires will be necessary to quantify the relative contribution of therapeutic success and sub-optimal therapeutic adherence to the low prevalence of ADRM observed in Roraima. Finally, information about previous and current ART regimens used by the patients will be also of paramount importance to fully explain the high rate of ADRM observed among patients with undetectable plasma viremia.

In conclusion, our study indicates that the HIV-1 epidemic in Roraima is mostly dominated by the B subtype and that the epidemiological relevance of other subtypes and recombinants forms is much lower than that described in other Brazilian states. Our findings also revealed a relatively high frequency of undetectable viral load and a relatively low prevalence of ADRM among treatment-experienced individuals from Roraima. As the HIV-1 epidemic and the use of ART spreads towards the innermost parts of Brazil, continued molecular surveillance studies will be essential to monitor temporal changes in the prevalence of drug resistant strains among treatment naïve and experienced patients in those areas.

## Supporting information

S1 Table Viral load, CD4 information and genbank access number of the sequences of each patient of HIV-1-infected patients attended at the Public Health Central Laboratory in Boa Vista, Roraima. (DOCX)
