# Supplementary material for: HIV-1 genetic diversity and antiretroviral drug resistance among individuals from Roraima state, northern Brazil
Source: PLoS One. 2017 Mar 16;12(3):e0173894. doi: 10.1371/journal.pone.0173894 (PMC5354385; doi:10.1371/journal.pone.0173894)
Supplement: S1 Table — (DOCX) [file pone.0173894.s001.docx]

**Supplementary table 1.** Viral load, CD4 information and genbank access number of the sequences of each patient of HIV-1-infected patients attended at the Public Health Central Laboratory in Boa Vista, Roraima.

| Patient ID | Accession  Number | | RNA Viral Load  (copies/ml) | | CD4+ T-cell counts  (cells/µl) |
| --- | --- | --- | --- | --- | --- |
| BR.RR.2013 MADS7 | | KX443070 | | 19.256 | 616 |
| BR.RR.2013 FMDS8 | | KX443038 | | <40 | 349 |
| BR.RR.2013 GKMM11 | | KX443045 | | 17.039 | 669 |
| BR.RR.2013 RLDMS12 | | KX443076 | | <40 | 367 |
| BR.RR.2013 SDS14 | | KX443081 | | <40 | 468 |
| BR.RR.2013 RMDS15 | | KX443078 | | <40 | 616 |
| BR.RR.2013 VADS16 | | KX443084 | | 5.478 | 217 |
| BR.RR.2013 ICAF18 | | KX443047 | | 9.057 | 604 |
| BR.RR.2013 MFBG21 | | KX443061 | | 362 | 601 |
| BR.RR.2013 CAT22 | | KX443024 | | 1.383.017 | 1042 |
| BR.RR.2013 ER 23 | | KX443031 | | 51 | 350 |
| BR.RR.2013 JNDAF27 | | KX443053 | | <40 | 255 |
| BR.RR.2013 MG29 | | KX443063 | | 1.364.348 | - |
| BR.RR.2013 RBM30 | | KX443074 | | <40 | 414 |
| BR.RR.2013 FGM31 | | KX443035 | | <40 | 427 |
| BR.RR.2013 FJF32 | | KX443037 | | <40 | 957 |
| BR.RR.2013 JDOG 35 | | KX443050 | | 857 | 227 |
| BR.RR.2013 LOBM 36 | | KX443057 | | <40 | 533 |
| BR.RR.2013 MRDS37 | | KX443066 | | 90 | 214 |
| BR.RR.2013 ABF38 | | KX443016 | | 748 | 258 |
| BR.RR.2013 MDCFC39 | | KX443060 | | <40 | 401 |
| BR.RR.2013 ERP41 | | KX443032 | | <40 | - |
| BR.RR.2013 ADNDS 43 | | KX443020 | | <40 | 629 |
| BR.RR.2013 AAV 49 | | KX443015 | | <40 | 720 |
| BR.RR.2013 VFDB 50 | | KX443086 | | 671.146 | 1822 |
| BR.RR.2013 FSDC 52 | | KX443043 | | - | - |
| BR.RR.2013 ACDC 56 | | KX443017 | | <40 | 658 |
| BR.RR.2013 MMDR 57 | | KX443065 | | 49.518 | 360 |
| BR.RR.2013 MADM 58 | | KX443059 | | 522 | 268 |
| BR.RR.2013 JLM 61 | | KX443051 | | <40 | 238 |
| BR.RR.2013 MFO 62 | | KX443062 | | <40 | 349 |
| BR.RR.2013 PWDS 63 | | KX443073 | | <40 | 557 |
| BR.RR.2013 CDSF 64 | | KX443023 | | <40 | 499 |
| BR.RR.2013 RFDS66 | | KX443075 | | 2.220 | 346 |
| BR.RR.2013 ALF 68 | | KX443021 | | <40 | 286 |
| BR.RR.2013JMNG69 | | KX443052 | | <40 | 518 |
| BR.RR.2013 VD71 | | KX443085 | | <40 | 487 |
| BR.RR.2013 ES 73 | | KX443033 | | <40 | 579 |
| BR.RR.2013 OVM76 | | KX443072 | | <40 | 285 |
| BR.RR.2013 RLT78 | | KX443077 | | <40 | 874 |
| BR.RR.2013 SA 79 | | KX443080 | | <40 | 614 |
| BR.RR.2013 JCDA 80 | | KX443049 | | 184 | 266 |
| BR.RR.2013 TDSA85 | | KX443083 | | <40 | 679 |
| BR.RR.2013 OSDM 86 | | KX443071 | | <40 | 266 |
| BR.RR.2013 EADS 87 | | KX443026 | | <40 | 631 |
| BR.RR.2013 LRD 88 | | KX443058 | | 3.277 | 123 |
| BR.RR.2013 HODA89 | | KX443046 | | <40 | 411 |
| BR.RR.2013 FDSS 93 | | KX443034 | | 277 | 422 |
| BR.RR.2013 ION 94 | | KX443048 | | 43.525 | 181 |
| BR.RR.2013 FPC 97 | | KX443040 | | <40 | 354 |
| BR.RR.2013 FSDC 99 | | KX443044 | | <40 | 568 |
| BR.RR.2013 DJSG 100 | | KX443025 | | <40 | 168 |
| BR.RR.2013 EDA 101 | | KX443028 | | 60 | 189 |
| BR.RR.2013 ELA 103 | | KX443029 | | <40 | 210 |
| BR.RR.2013 SNT104 | | KX443082 | | <40 | 464 |
| BR.RR.2013 ECV 106 | | KX443027 | | <40 | 382 |
| BR.RR.2013 JNDS107 | | KX443054 | | <40 | 502 |
| BR.RR.2013 ELR401 | | KX443030 | | - | - |
| BR.RR.2013 RP403 | | KX443079 | | - | - |
| BR.RR.2013 NADS404 | | KX443069 | | <40 | 189 |
| BR.RR.2013 WSDA 407 | | KX443087 | | 90 | 588 |
| BR.RR.2013 FS 408 | | KX443042 | | 49.856 | 380 |
| BR.RR.2013 MRDS 409 | | KX443067 | | <40 | 616 |
| BR.RR.2013 ACO 410 | | KX443018 | | 18.612 | 272 |
| BR.RR.2013 KAL411 | | KX443056 | | 15.919 | 338 |
| BR.RR.2013 ML413 | | KX443064 | | <40 | 277 |
| BR.RR.2013 AS 415 | | KX443022 | | - | - |
| BR.RR.2013 JR 418 | | KX443055 | | <40 | 613 |
| BR.RR.2013 FRD419 | | KX443041 | | - | - |
| BR.RR.2013 FGS 420 | | KX443036 | | <40 | 186 |
| BR.RR.2013 MS421 | | KX443068 | | 14.270 | 411 |
| BR.RR.2013 ACVF 423 | | KX443019 | | 423.06 | 151 |
| BR.RR.2013 FMS 425 | | KX443039 | | <40 | 74 |
